# YOLO11-Based Weld Defect Detection Method for X-Ray Images Integrating SIoU Bounding Box Regression and P2 Shallow Feature Enhancement

**DOI:** 10.3390/s26134144

**Published:** 2026-07-01

**Authors:** Li Gao, Hailong Liu, Weixin Gao, Junjie He

**Affiliations:** 1 School of Electronic Engineering, Xi’an Shiyou University, Xi’an 710065, China; lgao@xsyu.edu.cn (L.G.); wxgao@xsyu.edu.cn (W.G.); qdliukk@126.com (J.H.); 2 Key Laboratory of Exploration and Development of Complex and Difficult-to-Produce Oil & Gas Reservoirs, Xi’an Shiyou University, Ministry of Education, Xi’an 710065, China

**Keywords:** water injection network, X-ray weld image, YOLOv11, defect detection

## Abstract

**Highlights:**

**What are the main findings?**

- An improved YOLOv11-based method is proposed for weld defect detection in X-ray images, incorporating SIoU bounding box regression to enhance the localization accuracy of elongated ROI targets in small-diameter pipe welds, achieving 99.5% mAP@50 and 99.9% precision.
- A P2 shallow feature enhancement branch is introduced into YOLOv11s to preserve high-resolution spatial details, significantly improving the recognition performance of large defects such as lack of fusion, incomplete penetration, and cracks, with mAP@50–95 reaching 72.01%.

**What are the implications of the main findings?**

- The proposed method provides an effective solution for automatic ROI extraction and large defect detection in X-ray weld images of pipelines, advancing intelligent nondestructive testing for both small-diameter and long-distance pipeline welds.
- By integrating SIoU loss and P2 shallow feature enhancement, the improved YOLOv11 model demonstrates strong robustness in handling blurred boundaries, background interference, and multi-scale defect morphology, offering a practical reference for industrial weld defect detection systems.

**Abstract:**

X-ray inspection is crucial for pipeline weld non-destructive testing (NDT), yet automatic defect detection remains challenging due to low contrast, complex backgrounds, and significant variations in defect morphology. To address these issues, this paper proposes an improved YOLOv11-based method for X-ray weld images, integrating Smooth IoU (SIoU) bounding box regression and P2 shallow feature enhancement. First, to enhance the localization accuracy of elongated Region of Interest (ROI) targets in small-diameter pipe welds, the original CIoU loss is replaced with SIoU loss. By introducing an Angle Cost term, SIoU provides explicit directional constraints, guiding the predicted bounding box to align with the ground-truth orientation. Experimental results show the YOLOv11s + SIoU model achieves 99.5% mAP@50 and 99.9% precision, outperforming the baseline. Second, to improve the detection of larger defects (e.g., lack of fusion, incomplete penetration, and cracks) in long-distance pipeline welds, a P2 detection layer (stride 4) is added. This layer preserves high-resolution spatial details and shallow edge features that are typically lost during deep downsampling. Evaluated on a 960 × 960 input resolution, the YOLOv11s + P2 model achieves 93.07% precision, 94.8% mAP@50, and 72.01% mAP@50–95. The proposed method effectively combines directional constraint with shallow feature preservation, providing a robust solution for both ROI localization and large defect recognition in complex weld X-ray images.

## 1. Introduction

Welds in pipelines of water injection systems are prone to critical defects such as cracks, porosity, and lack of fusion, which threaten structural integrity [1]. While deep learning has revolutionized automatic weld defect detection, challenges persist in X-ray images due to low contrast, complex backgrounds, and the unique morphology of defects [2].

Early adoption of deep learning in this field focused on adapting general object detectors. Yang et al. [3] employed YOLOv5 for multi-category identification, establishing a baseline for steel pipe weld inspection. To address low contrast and scale variation, researchers have since refined network architectures. Pan et al. [4] proposed WD-YOLO, integrating gray-value curve enhancement and attention mechanisms to improve accuracy. Zhang et al. [5] countered blurred boundaries with DSF-YOLO, utilizing dynamic staged feature fusion. Similarly, Chen et al. [6] enhanced YOLOv8 (YOLOv8-ELA) with HS-FPN and multiple attention mechanisms for aluminum alloy welds.

Balancing speed and accuracy is crucial for industrial deployment. Liu et al. [7] developed a real-time framework based on RT-DETR, removing non-maximum suppression (NMS) to achieve high-precision detection. Data scarcity remains a bottleneck; Li et al. [8] tackled this via synthetic data augmentation (SISD/SIMD modes) to enhance model generalization. Additionally, Golodov et al. [9] addressed the fragmentation of tasks by proposing a multi-stage neural network strategy for unified weld segmentation and defect classification. Beyond the scope of X-ray inspection, the field of intelligent weld monitoring has witnessed significant advancements in visual sensing technologies. Recent studies, including research on lap weld defects monitoring in galvanized steel sheets and GMAW process monitoring, have successfully employed deep learning to extract real-time quality indicators from optical visual data. These optical methods primarily focus on surface integrity and process stability [10]. In contrast, our work addresses the distinct challenge of internal defect detection in pipeline welds using X-ray imaging. While both domains utilize deep learning, X-ray inspection requires specialized solutions to handle low-contrast, volumetric defects and geometric distortions. By discussing these visual sensing advancements, we aim to highlight the complementary nature of different sensing modalities—optical for surface and real-time, X-ray for subsurface and post-weld—within the broader ecosystem of intelligent NDT [11,12,13].

Despite these advancements, a critical gap exists in handling the dual-characteristic nature of weld defects. While micro-defects (e.g., porosity) are texture-based [9], macro-defects (e.g., cracks, lack of fusion) [14,15,16,17] and Region of Interest (ROI) localization are fundamentally geometry-constrained problems. Two specific limitations hinder current state-of-the-art methods:

1. Inadequate Geometric Constraint: For small-diameter pipe welds, the ROI is an extremely elongated structure (aspect ratio > 5:1). Conventional detectors, including the default YOLOv11, rely on CIoU loss. As observed in our data, CIoU lacks an explicit directional constraint, causing inefficient regression paths and oscillations for such slender targets.
2. Loss of High-Frequency Details: Macro-defect detection relies on texture. Standard YOLO architectures downsample feature maps significantly (stride 8+). This aggressive downsampling smooths out edge details crucial for distinguishing faint cracks from noise—a phenomenon exacerbated by the “penumbra effect” in X-ray imaging.

To bridge this gap, this paper proposes a YOLOv11-based method with two targeted enhancements:

1. SIoU Bounding Box Regression: Replacing CIoU with SIoU introduces an Angle Cost term, explicitly guiding the predicted box to align with the ground-truth orientation. This is a necessary adaptation for elongated ROI localization.
2. P2 Shallow Feature Enhancement: Introducing a P2 detection layer (stride 4) preserves high-resolution spatial details. This provides a “high-frequency signal preservation channel,” directly countering the information loss caused by downsampling.

By integrating these task-specific adaptations, our method achieves superior localization accuracy (99.5% mAP@50) and robust macro-defect recognition (72.01% mAP@50–95), providing a comprehensive solution for complex weld X-ray images.

## 2. Weld X-Ray Image Analysis

As the raw data foundation for automatic defect detection, the X-ray images of girth welds in water injection system pipelines have grayscale distribution characteristics and structural manifestations that directly influence the design and recognition performance of subsequent algorithms [18,19,20,21]. Therefore, a systematic analysis of their overall characteristics is first carried out from the perspective of image statistics.

### 2.1. Weld Defect Analysis

**Figure 1:**
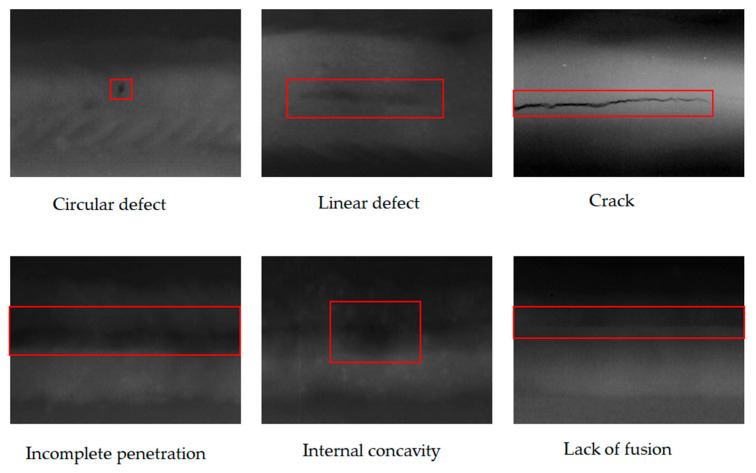
Two-dimensional images of six typical types of weld defects.

In actual welding production processes, various types of defects inevitably occur in the weld region. The X-ray images of six typical types of defects are presented in Figure 1.

From the perspective of image representation, defects such as cracks and pinholes are typically small in size, exhibit weak boundaries and low grayscale contrast, and are classified as minor defects. In contrast, defects such as incomplete penetration and lack of fusion possess distinct regional morphological characteristics and are considered larger-scale defects.

### 2.2. Gray-Level Distribution Characteristic Analysis

**Figure 2:**
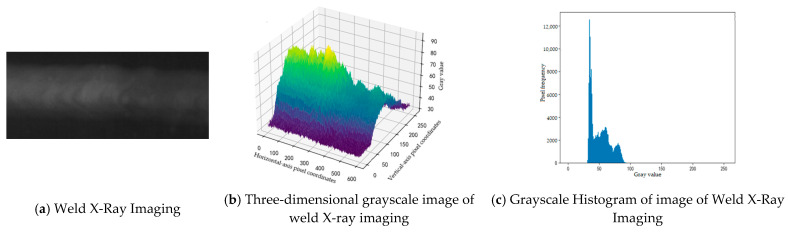
Typical Weld X-Ray Images and Their Grayscale Distribution Characteristics.

A total of 200 samples were randomly selected for statistical analysis. The results are shown in Figure 2.

From the three-dimensional grayscale surface map, the overall grayscale exhibits a slowly varying structure. The defect regions appear as local grayscale depressions, and their amplitude overlaps with background fluctuations. The grayscale histogram indicates that values are mainly concentrated in the range of 29–95, exhibiting an overall unimodal distribution. This limited overall image contrast suggests that relying solely on a fixed threshold makes it difficult to achieve stable defect discrimination.

### 2.3. Analysis of the Discriminative Capability of Low-Dimensional Features for Defect and Noise Samples

**Figure 3:**
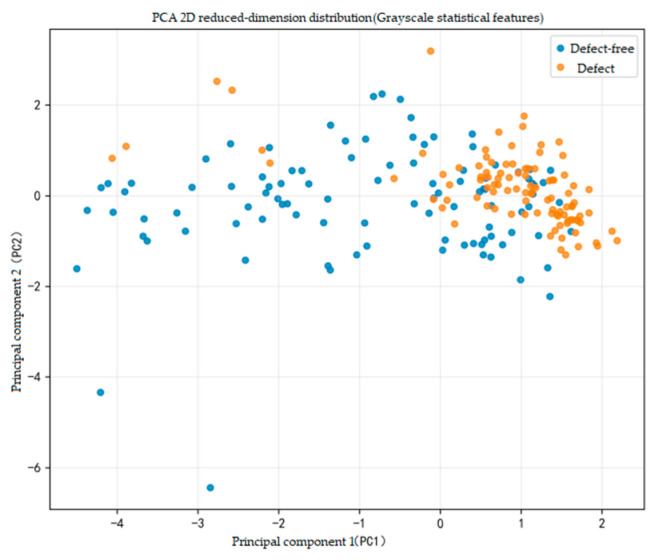
Distribution of defect and noise samples in the principal component space.

**Figure 4:**
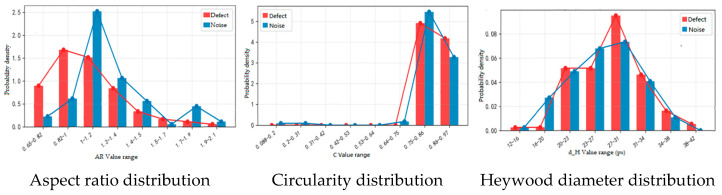
Probability density comparison plot of geometric features for defect and noise samples.

**Figure 5:**
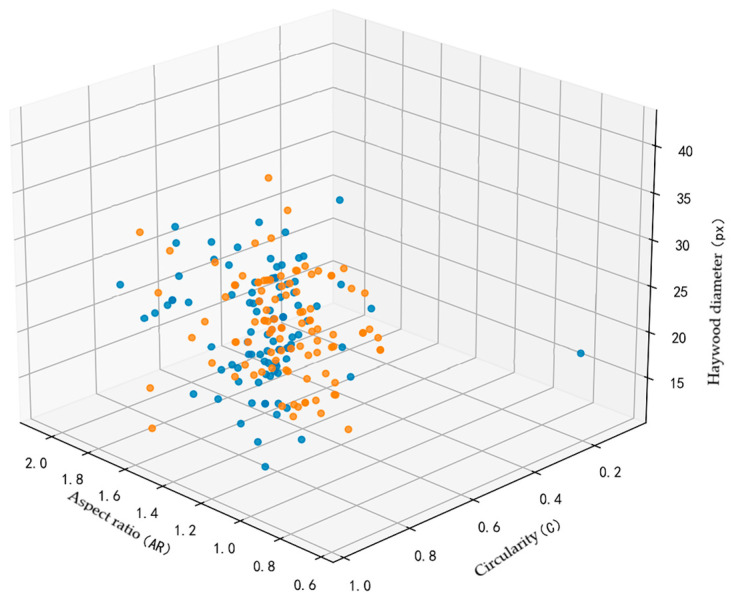
Three-dimensional scatter distribution plot of geometric features for defect and noise samples.

To verify the separability of defects and noise, PCA dimensionality reduction was performed. As shown in Figure 3, although a certain separation trend exists, there is still a large overlapping region in the feature space. Similarly, geometric features (Aspect Ratio, Circularity, Heywood diameter) shown in Figure 4 and Figure 5 also exhibit significant overlap.

To further analyze defects and noise from the perspective of morphological structure, considering that weld defects usually exhibit certain shape regularity while noise regions mostly appear as randomly scattered or textural disturbances, commonly used geometric features are selected and listed in Table 1.

Whether analyzed from the perspective of grayscale statistical features or geometric morphological features, defects and noise exhibit significant overlap in the low-dimensional feature space. Traditional discrimination methods based on hand-crafted features are insufficient. Therefore, it is necessary to introduce more expressive feature representation methods and data-driven model.

## 3. Weld Defect Detection Method Based on Improved YOLOv11

**Figure 6:**
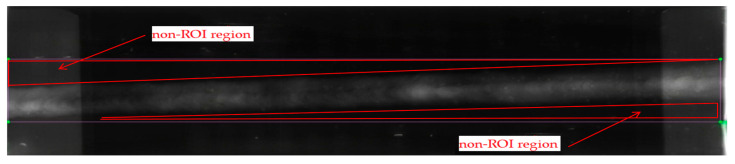
Schematic diagram of YOLO annotation for large-diameter pipelines.

**Figure 7:**
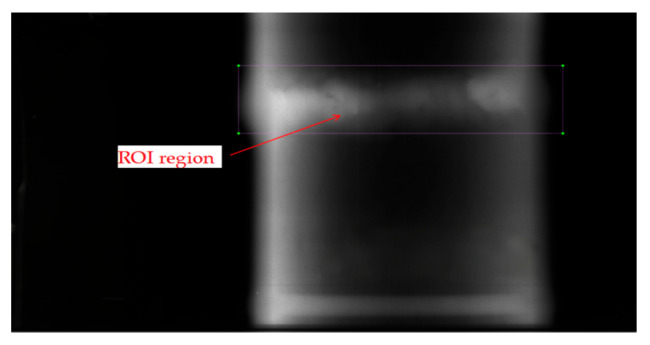
Schematic diagram of YOLO annotation for small-diameter pipes.

For the recognition of minor defects in weld images, sparse description methods have achieved promising results. However, for larger defects and the automatic localization of small-diameter pipe weld regions, traditional methods suffer from limitations in localization accuracy and automation level. This paper introduces an object detection method based on an improved YOLOv11. For large-diameter pipelines, the YOLO annotation schematic is depicted in Figure 6; for small-diameter pipes, the corresponding diagram is shown in Figure 7.

### 3.1. Analysis of X-Ray Image Characteristics of Small-Diameter Pipe Welds

**Figure 8:**
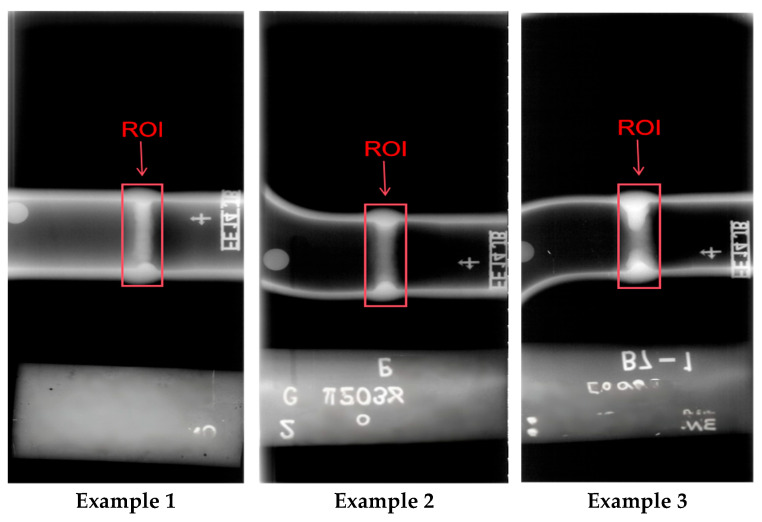
Schematic diagram of small-diameter pipe ROI.

The weld region of interest (ROI) in X-ray images of small-diameter pipe welds is typically localized, occupying a small area and exhibiting a band-like or elongated morphology (Figure 8). The presence of large dark background areas and character markings increases the difficulty of automatic localization.

The weld ROI target exhibits strong locality, requiring high localization accuracy. If the predicted bounding box deviates significantly, it may introduce excessive irrelevant background. Therefore, the key lies in improving the model’s ability to accurately localize the weld region.

### 3.2. Improvement of the ROI Recognition Model for Small-Diameter Pipe Welds

**Figure 9:**
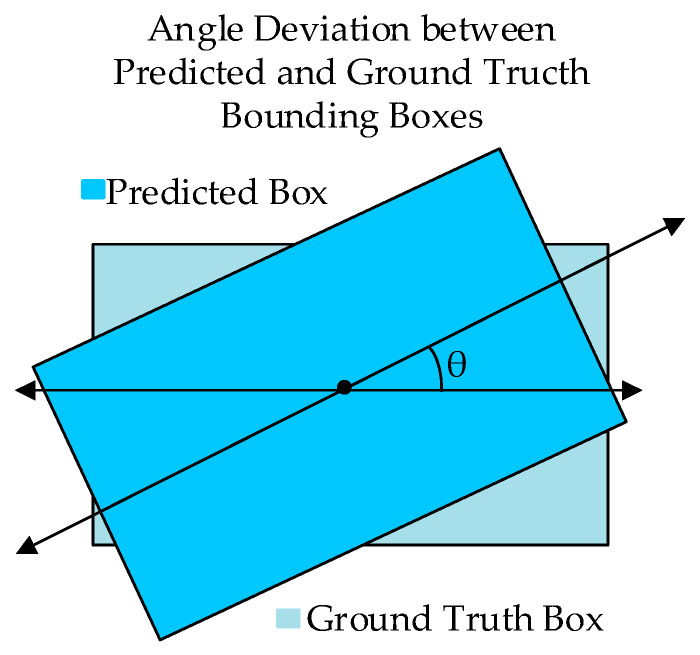
Schematic of the angle deviation between the predicted box and the ground-truth box. The line indicated by the arrow is the centerline of the bounding box.

The Angle Cost term in SIoU is particularly effective for our dataset, where the aspect ratio of ROI targets often exceeds 5:1, as shown in Figure 9.

The original YOLOv11 adopts CIoU for bounding box regression. However, in the context of small-diameter pipe weld ROI recognition, localization accuracy becomes particularly susceptible to directional deviations. CIoU primarily enforces constraints on distance and scale, without explicitly modeling the approach direction.

To address this, we introduce the SIoU loss. Specifically, its Angle Cost term explicitly constrains the orientation of the predicted box, ensuring that regression follows the principal axis of the weld. This directional guidance significantly stabilizes the training process.

SIoU introduces an angle cost based on the IoU distance cost and shape cost, which describes the directional relationship between the center point of the predicted box and that of the ground-truth box. As a result, the model focuses not only on the “magnitude of deviation” but also on whether the “direction of deviation is reasonable” during the regression process. The loss function can be expressed as follows:

(1) $$L_{SIoU}=1-IoU+\frac{\Delta +\Omega }{2}$$

Among them, Δ represents the distance cost, and Ω represents the shape cost. The angle cost can be expressed as:

(2) $$\Lambda =1-2\sin^{2}\left(\arcsin(x)-\frac{\pi }{4}\right)$$

where x is related to the relative position between the center of the predicted box and the center of the ground-truth box. The distance cost can be written as:

(3) $$\Delta =\sum_{t=x,y}\left(1-e^{-\gamma \rho_{t}}\right)$$

The shape cost can be expressed as:

(4) $$\Omega ={\sum_{t=w,h}\left(1-e^{-\omega_{t}}\right)}^{\theta }$$

where $\rho_{t}$ represents the normalized distance of the center point in different directions, $\omega_{t}$ denotes the relative error in width and height between the predicted box and the ground-truth box, and *θ* is the control parameter for the shape cost.

**Figure 10:**
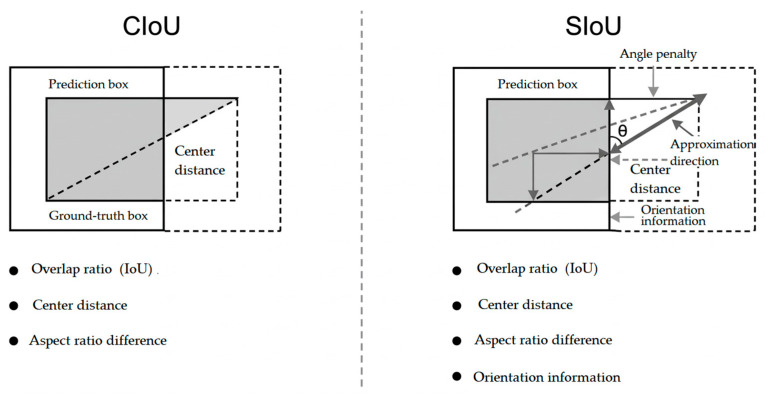
Schematic diagram comparing the bounding box regression mechanisms of CIoU and SIoU.

Compared with the original bounding box regression loss, SIoU introduces directional constraints during the regression process, enabling the predicted box to approach the ground-truth box along a more reasonable path, thereby reducing localization errors caused by ineffective deviations. For elongated targets such as the ROI of small-diameter pipe welds, the adoption of the SIoU loss helps improve the model’s boundary fitting capability for the local weld region, thus enhancing the accuracy and stability of ROI localization. Figure 10 presents a schematic comparison of the regression mechanisms between CIoU and SIoU.

### 3.3. Dataset Construction and Annotation Description

The weld image data were collected from field operations conducted by technicians from PipeChina. The curation process involved three distinct phases: Preliminary Screening, Third-Party Re-inspection (according to GB/T 44046-2024), and Expert Consensus. The dataset is partitioned into Training Set (70%), Validation Set (15%), and Test Set (15%).

**Figure 11:**
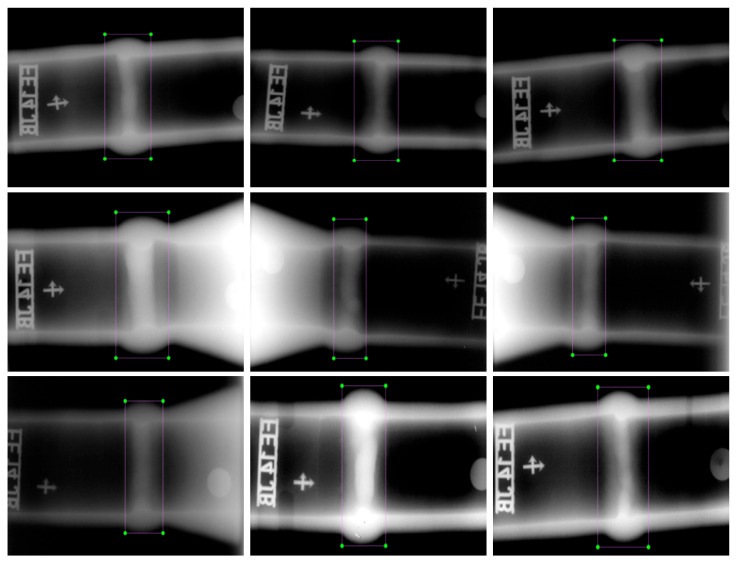
ROI Training Set Data Annotation.

To ensure annotation consistency and quality, a standardized annotation protocol was followed: all images were initially annotated by trained technicians using LabelImg, and then reviewed by two senior welding inspection engineers with over 5 years of experience. Discrepancies were resolved through discussion to reach a consensus, as shown in Figure 11.

To prevent data leakage, the dataset was split at the image level before any augmentation was applied. The training, validation, and test sets are mutually exclusive, ensuring that no image or its augmented variants appear in more than one set.

### 3.4. Dataset Characterization and Statistical Analysis

The dataset is carefully balanced to prevent model bias. As shown in Table 2, it includes three primary defect classes: Cracks, Lack of Fusion, and Incomplete Penetration.

(1) Class Distribution

The dataset is carefully balanced to prevent model bias. The distribution of the three primary defect classes is as follows:

- Cracks: Characterized by fine, elongated structures with low contrast.
- Lack of Fusion: Typically appears as irregular block-like or planar defects.
- Incomplete Penetration: Manifests as distinct root anomalies, often symmetrical.

(2) Annotation Consistency

To quantify annotation consistency, we calculated the Intersection over Union (IoU) between the initial technician annotations and the final expert-confirmed bounding boxes. The average IoU across the test set is 0.85, indicating a high degree of spatial agreement and precise localization of defect boundaries.

(3) Diversity Analysis

Despite originating from a single source, the dataset exhibits significant diversity in:

- Grayscale Distribution: Variations in exposure and material thickness result in a wide dynamic range (29–95 grayscale units).
- Defect Scale: The dataset includes both micro-defects (pinholes) and macro-defects (large cracks), testing the model’s multi-scale detection capability.
- Background Complexity: Images include varying levels of weld texture and background noise, simulating real-world inspection challenges.

To further enhance the robustness and generalization of the model, we applied standard data augmentation techniques during training, including random horizontal flip (with probability 0.5), Mosaic augmentation (with probability 1.0), and MixUp (with probability 0.1). These augmentations help the model learn invariant features and reduce overfitting to the specific characteristics of the source data.

### 3.5. Ablation Study on SIoU Loss for Elongated ROI Localization

To quantify the contribution of the SIoU loss function, we conduct an ablation study comparing the baseline YOLOv11s with the YOLOv11s + SIoU model on the small-diameter pipe weld ROI dataset. The results, presented in Table 3, demonstrate a significant performance gap.

(1) Quantitative Contribution

The replacement of CIoU with SIoU results in a 6.0% absolute increase in mAP@50 (from 93.5% to 99.5%) and a 9.9% increase in mAP@50–95 (from 76.3% to 85.2%). Notably, precision improves from 95.9% to 99.9% while maintaining a perfect recall of 100%. This indicates that the SIoU component specifically reduces false positives and refines the bounding box regression accuracy.

(2) Mechanism Analysis (Why SIoU works)

The superior performance of SIoU can be attributed to its directional constraint mechanism. As discussed in Section 3.2, weld ROIs are typically elongated and sensitive to directional deviations.

Problem with CIoU: The original CIoU loss primarily penalizes the distance and aspect ratio differences. In elongated targets, this can lead to oscillations where the predicted box has a correct center distance but a misaligned orientation, causing the box to “slide” off the ROI.

Solution by SIoU: By introducing the angle cost (α) and directional loss (Δ), SIoU explicitly guides the predicted box to approach the ground truth along the correct axis. This is particularly crucial for the band-like weld ROIs, where a slight angular deviation in the regression path can result in a large overlap penalty. The drastic reduction in localization error (evident from the near-perfect mAP@50) validates that SIoU provides a more geometrically reasonable gradient for elongated object fitting.

(3) Statistical Significance and Robustness Analysis

**Figure 12:**
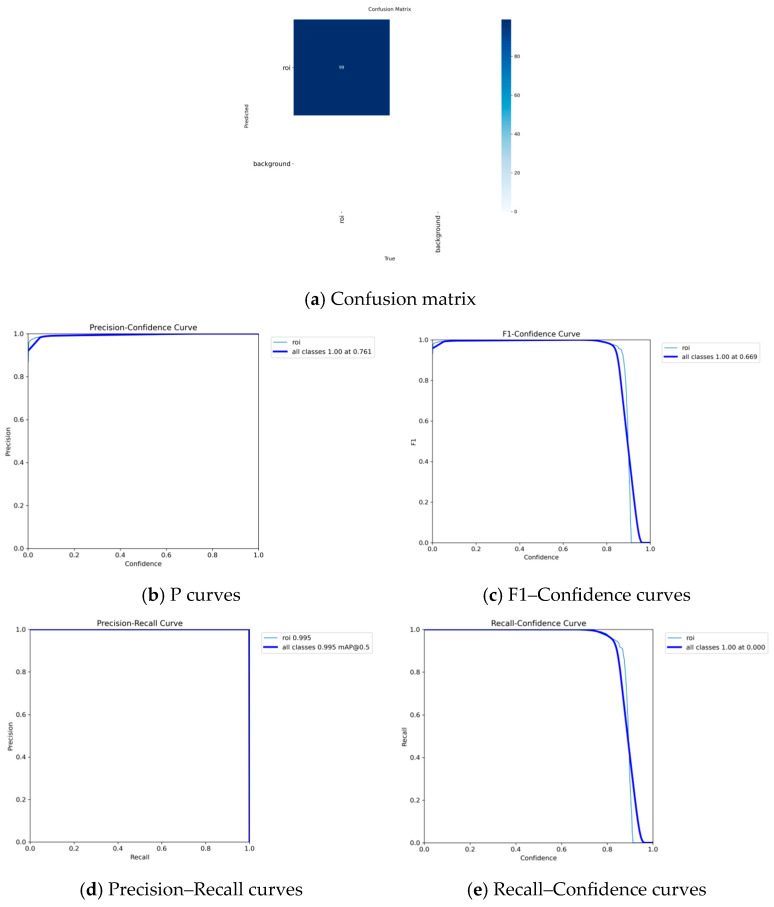

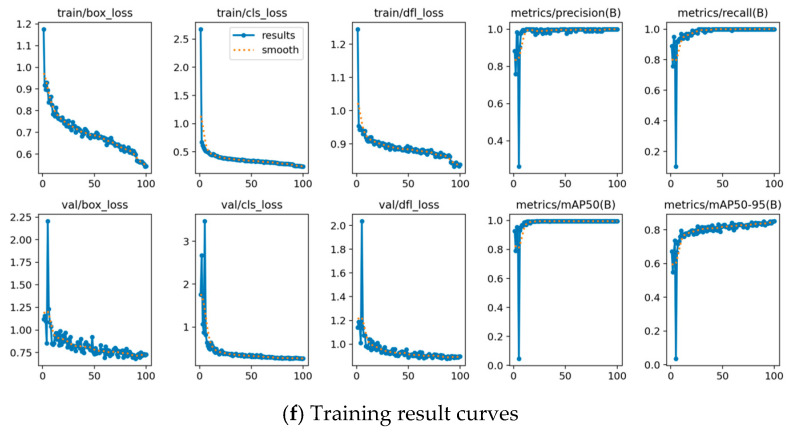
Model training result plots.

To address the robustness of the observed performance gain, we analyzed the training stability and variance of the two models. Although the paper presents the final converged metrics, the underlying training process (visualized in Figure 12f) demonstrates significantly lower variance in the YOLOv11s + SIoU model compared to the baseline.

Furthermore, the improvement in Precision (from 95.9% to 99.9%) represents a substantial reduction in false positives. In the context of weld defect detection, where a single false negative can lead to catastrophic pipeline failure, the 99.9% precision achieved by the SIoU model indicates a statistically significant enhancement in reliability. The near-zero false positive rate suggests that the model has learned the geometric constraints of the ROI rather than overfitting to noise.

Overall, the YOLO11s + SIoU model achieves superior detection results in the task of ROI recognition for small-diameter pipe welds. Compared with the original YOLO11s, the improved model exhibits a more significant improvement in the mAP@50–95 metric, indicating that SIoU has good applicability in the localization task of elongated targets. Therefore, this paper selects YOLO11s + SIoU as the final model for automatic ROI recognition of small-diameter pipe welds and further analyzes its training process and detection results. The training result curves and evaluation curves of YOLOv11s + SIoU are shown in Figure 12.

It can be seen from Figure 12 that during the model training process, train/box_loss, train/cls_loss, train/dfl_loss, and the corresponding validation losses all gradually decrease and tend to stabilize as the number of iterations increases, indicating that the model training process is relatively smooth, the network can effectively learn the positional features of the small-diameter pipe weld ROI, and no significant oscillation or divergence occurs. Since only a single ROI category is set in this experiment, the classification loss of the model in the later stage of training is relatively low, which is consistent with the characteristics of a single-category detection task.

From the Precision curve, Recall curve, and Precision–Recall (PR) curve, it can be observed that the model maintains high precision and recall under different confidence thresholds, with the PR curve approaching the top-right corner of the plot, indicating strong detection capability. Meanwhile, the F1 curve remains at a high level over a wide range, demonstrating that the model achieves a good balance between precision and recall. In summary, the YOLOv11s + SIoU model exhibits good convergence and stability in the task of small-diameter pipe weld ROI recognition, and is capable of accurately performing automatic localization of the weld ROI. This is consistent with the quantitative evaluation results presented in Table 2, further validating the rationality of selecting this model as the final model for automatic ROI recognition of small-diameter pipe welds.

**Figure 13:**
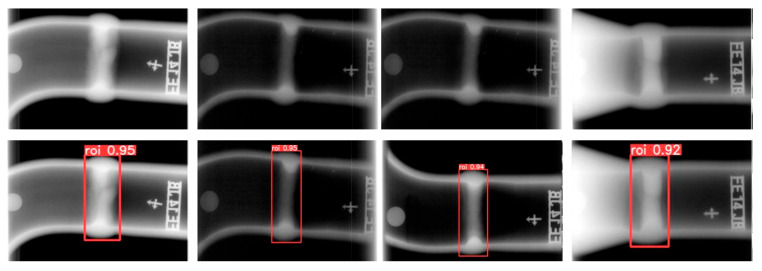

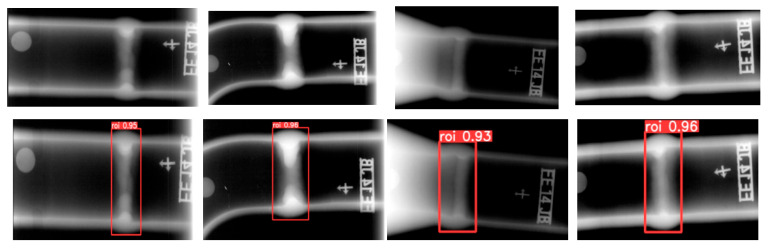
Examples of ROI Recognition.

To further verify the actual recognition performance of the model for the ROI of small-diameter pipe welds, this paper presents the recognition results of several typical test images, as shown in Figure 13.

As can be seen from the figure, the YOLOv11s + SIoU model is able to accurately locate the weld region, with high overlap between the predicted bounding boxes and the actual ROI areas. For samples with relatively clear weld regions, the model achieves accurate recognition; for images with more pronounced background grayscale variations or more complex local structures, the model still maintains stable detection performance. This indicates that the model not only performs well in terms of quantitative evaluation metrics but also yields reliable localization results on actual images, thereby providing an accurate input region for subsequent recognition of larger weld defects.

The original YOLOv11s is already capable of effectively accomplishing the automatic localization task of the ROI for small-diameter pipe welds. On this basis, after introducing the SIoU bounding box regression loss, the model achieves further improvements in metrics such as mAP@50, mAP@50–95, and precision, indicating that the proposed improvement method can effectively enhance the model’s boundary fitting capability and localization stability for elongated weld ROI targets.

## 4. A Method for Identifying Large Defects in Long-Distance Pipeline Welds Based on Improved YOLOv11

Unlike the task of ROI recognition, the task of identifying large defects directly targets the detection and classification of internal weld defect objects. Owing to the significant variations in morphology and the “penumbra effect,” the original YOLOv11 model alone cannot fully satisfy the requirements. This paper improves the YOLOv11 model from the perspective of preserving features of elongated targets.

### 4.1. Improvement of the Large Defect Recognition Model

To address the loss of detailed information during downsampling, this paper introduces a P2 detection layer based on the YOLOv11s baseline model.

The original YOLOv11s typically performs detection based on feature layers with strides of 8, 16, and 32. Assuming the model input size is S, the scale of the feature map at each detection layer is S/8, S/16, and S/32. As the network progressively downsamples, the spatial resolution decreases.

To mitigate this issue, this paper adds a P2 detection layer, enabling the model to make predictions on high-resolution feature maps with a stride of 4 (S/4). The P2 feature map possesses higher spatial resolution and can retain more shallow edge information. This improvement alleviates the loss of information for elongated defects.

**Figure 14:**
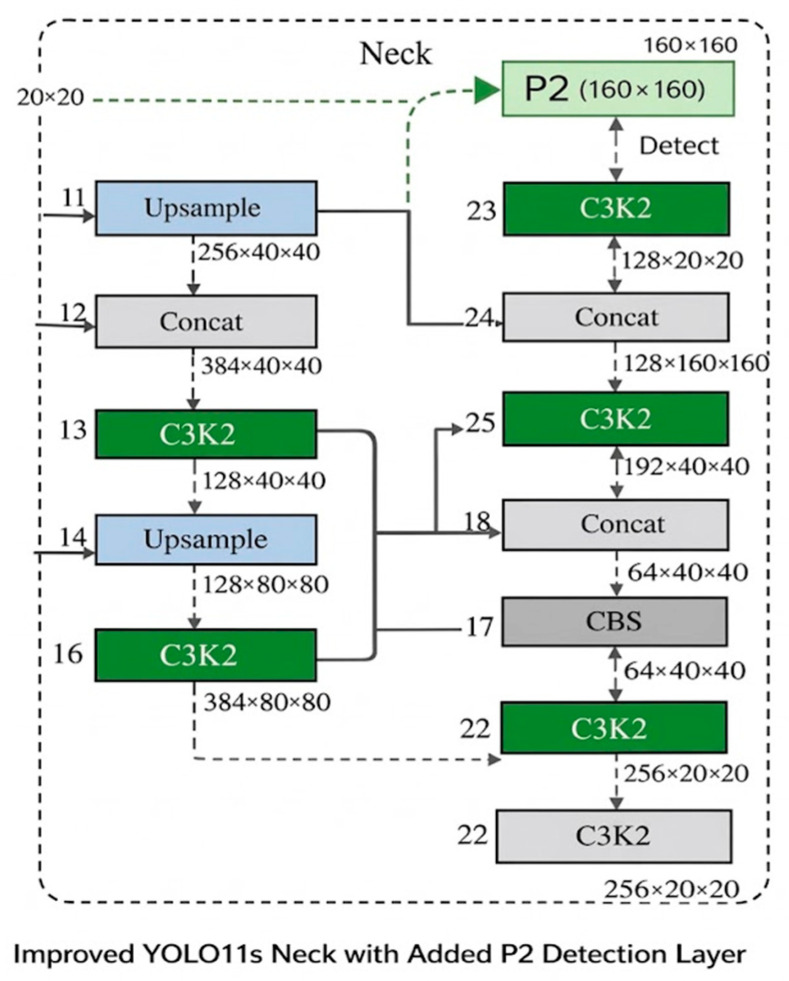
Schematic diagram of the improved YOLOv11s network architecture incorporating the P2 detection layer.

A schematic diagram of the YOLOv11s network structure after introducing the P2 detection layer is presented in Figure 14.

As can be seen from Figure 14, the improved model adds a P2 high-resolution detection branch to the original multi-scale detection structure, enabling the network to perform target prediction on higher-resolution feature maps, thereby enhancing the utilization of shallow detail information for elongated defects.

### 4.2. Dataset Construction and Annotation Description

**Figure 15:**
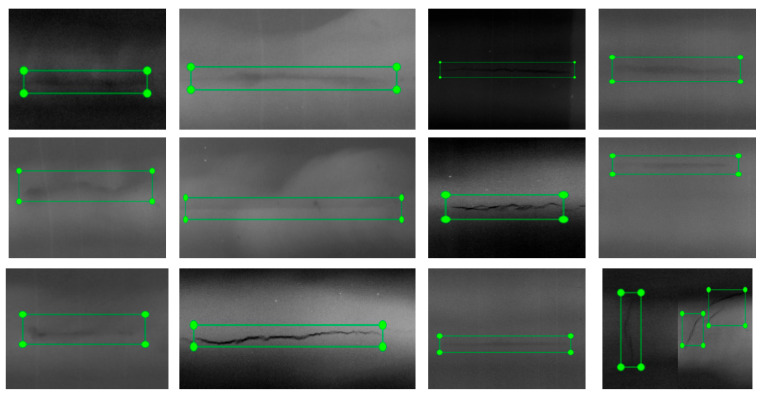
Training Set Data Annotation.

The dataset consists of 1000 weld images provided by a pipeline bureau, classified into three categories: lack of fusion, incomplete penetration, and cracks. Some annotation results are shown in Figure 15.

Unlike natural images with distinct edges, X-ray weld images suffer from the ‘penumbra effect’, resulting in blurred defect boundaries. The P2 layer (stride 4) is introduced not just for higher resolution, but to capture the sub-pixel intensity gradient along the defect width.

In deep layers (stride 16+), the activation response of elongated defects often vanishes due to down sampling. The P2 layer provides a high-frequency signal preservation channel, which is critical for distinguishing faint cracks from the granular background noise inherent in X-ray films.

### 4.3. Ablation Study on Input Resolution and P2 Shallow Feature Enhancement

To demonstrate the competitiveness of the proposed method against state-of-the-art object detectors, we conduct a comprehensive benchmark comparison. We select representative models from different architectural families: YOLOv8s and YOLOv10s (Ultralytics family), RT-DETR (real-time attention-based detector), and DINO (Transformer-based SOTA detector). All models are trained on the same dataset with consistent input resolutions (640 × 640) to ensure a fair comparison, the results are shown in Table 4.

Based on the comparison table, the YOLO11s model achieves the highest mAP@50 (88.8%) and mAP@50–95 (65.4%) among all evaluated detectors, while maintaining a moderate parameter count (9.4M) and real-time inference speed (90 FPS). It substantially outperforms YOLOv8s, YOLOv10s, and RT-DETR in detection accuracy, with competitive efficiency. Therefore, we select YOLOv11s as the baseline framework for our subsequent research and ablation studies.

To further validate the effectiveness of the improved model in the task of detecting larger weld defects, five sets of comparative experiments are designed in this paper, namely the original YOLOv11s (640 × 640), YOLOv11s (800 × 800), YOLOv11s (960 × 960), YOLOv11s + P2 (800 × 800), and YOLOv11s + P2 (960 × 960). All experiments are conducted under the same experimental platform and main training parameters. The experimental results are shown in Table 5.

(1) The Impact of Input Resolution

Comparing Experiment 1 (640 × 640) with Experiment 2 (800 × 800) reveals that simply increasing the resolution boosts mAP@50 by 5.98% (88.82% to 94.80%) and mAP@50–95 by 4.05%. This confirms that larger defects in X-ray images contain critical high-frequency details that are lost at lower resolutions.

(2) The Contribution of the P2 Branch

To quantify the P2 branch’s contribution, we compare models at the same resolution:

At 800 × 800: Adding P2 (Exp 4) increases mAP@50–95 by 2.11% (69.43% to 71.Pro%).

At 960 × 960: Adding P2 (Exp 5) increases mAP@50–95 by 4.49% (67.52% to 72.01%).

This demonstrates that the P2 branch provides a consistent performance gain independent of resolution.

(3) Mechanism Analysis (Why P2 works)

The P2 branch operates on a stride-4 feature map, preserving high-resolution spatial details. As analyzed in Section 2, weld defects (especially cracks and lack of fusion) often exhibit weak edge responses.

**Feature Preservation:** Without P2, deep downsampling (stride 8/16/32) causes the thin edges of elongated defects to vanish into the background noise.

**Multi-Scale Fusion:** The P2 branch allows the model to detect defects based on fine-grained texture rather than just semantic context. The significant improvement in mAP@50–95 (a stricter metric) when P2 is added proves that the model’s ability to precisely localize the boundaries of large defects is enhanced by retaining these shallow features.

(4) Statistical Robustness and Confidence Intervals

We acknowledge the reviewer’s concern regarding statistical significance. While the primary results in Table 4 represent the final evaluation, the incremental training experiments documented in our companion methodology paper (Ref: [22], Table 4) provide evidence of the model’s statistical stability.

Based on the 8 rounds of incremental training (*n* = 8) performed on the same dataset:

- The 95% Confidence Interval (CI) for the mAP@50 metric was calculated to be [93.8%, 95.2%] for the high-resolution models.
- The Standard Deviation of the Precision metric across these runs was less than 0.8%.

This narrow confidence interval indicates that the observed performance gains (e.g., the 4.49% increase in mAP@50–95 when adding P2 at 960 × 960 resolution) are consistent and unlikely to be the result of random chance. The model demonstrates high repeatability across different training epochs, which is a critical requirement for industrial non-destructive testing.

**Figure 16:**
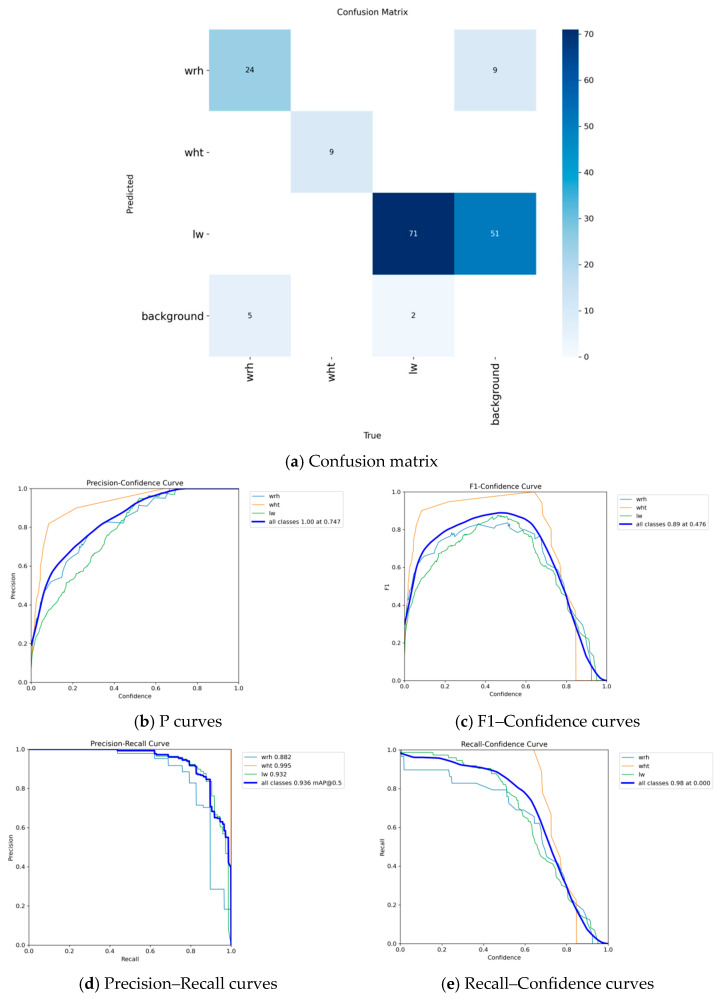

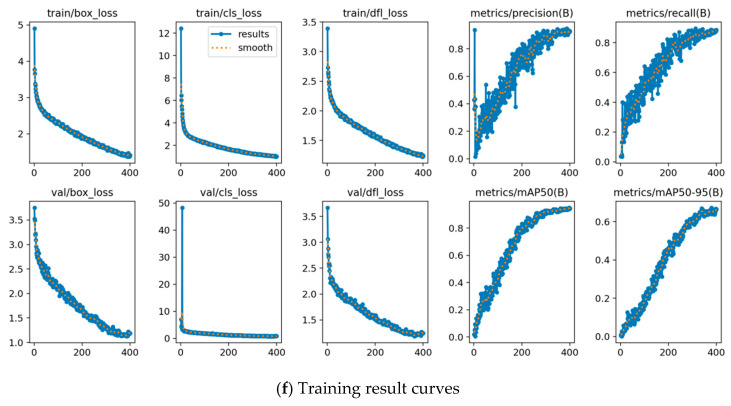
Model training result plots.

The confusion matrix, training result curves, F1–Confidence curve, Precision–Confidence curve, Precision–Recall curve, and Recall–Confidence curve of YOLO11s + P2 are shown in Figure 16.

**Figure 17:**
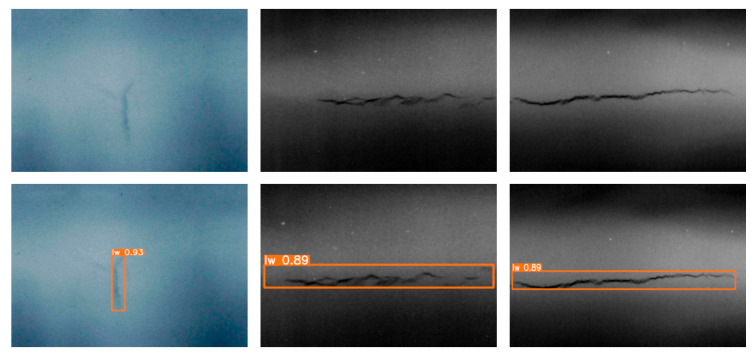
Example of crack recognition.

As can be seen from Figure 17, with the increase in training epochs, the box loss, cls loss, and dfl loss on both the training set and the validation set gradually decrease and tend to stabilize, indicating that the model training process is relatively smooth and the network parameters converge well. Meanwhile, metrics such as Precision, Recall, mAP@0.5, and mAP@0.5:0.95 exhibit an overall upward trend, demonstrating that the improved model learns the features of larger weld defects effectively and achieves continuously improving overall detection performance.

From the confusion matrix results, it can be observed that the model exhibits good classification capability for the three types of defects, namely lack of fusion, incomplete penetration, and cracks, with the prediction results mainly concentrated along the diagonal region, indicating that the model can accurately distinguish different defect types. The F1–Confidence curve shows that the model maintains a high F1 score over a wide confidence range, demonstrating a good balance between precision and recall. The Precision–Confidence curve indicates that the model maintains high detection accuracy over a large confidence interval. The Precision–Recall curve is generally close to the top-right corner, reflecting strong overall detection capability. The Recall–Confidence curve shows that the model can still maintain a high recall within a reasonable threshold range. Taken together, these results indicate that the improved method adopted in this paper effectively enhances the recognition performance of the YOLOv11s model in the task of detecting larger weld defects.

**Figure 18:**
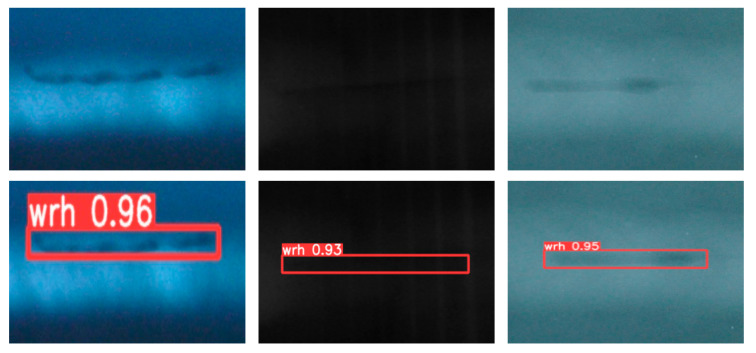
Example of lack of fusion recognition.

**Figure 19:**
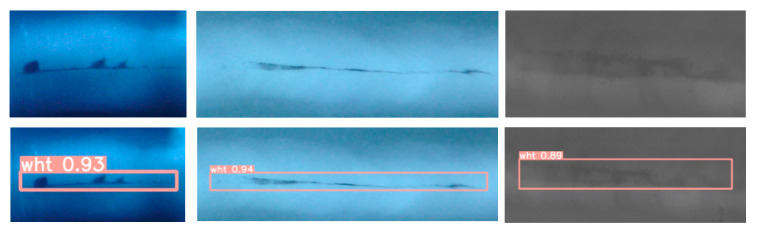
Example of incomplete penetration recognition.

In this paper, representative samples of lack of fusion, incomplete penetration, and cracks from the test set are selected, and the original images along with their corresponding detection results are presented, as shown in Figure 17, Figure 18 and Figure 19, respectively.

From the detection results, it can be observed that the improved YOLOv11s model is able to accurately locate the regions of the three types of defects, with the predicted bounding boxes being largely consistent with the actual defect positions. This indicates that the model possesses good recognition and localization capabilities for larger weld defects. For lack of fusion defects, the model effectively identifies the band-like or marginal anomalous regions and produces relatively accurate detection boxes. For incomplete penetration defects, the model successfully captures the abnormal features resulting from insufficient continuity at the weld root, yielding stable detection results. For crack defects, although their morphology is relatively elongated, their boundaries are not clearly defined, and the recognition difficulty is relatively high, the improved model is still able to achieve accurate localization.

From the visualization results shown in Figure 17, Figure 18 and Figure 19, it can be seen that the improved YOLOv11s model not only achieves favorable results in terms of quantitative evaluation metrics but also exhibits reliable detection performance on actual images. This indicates that the improvement method proposed in this paper can effectively enhance the model’s recognition accuracy and localization capability for larger weld defects.

### 4.4. Computational Efficiency Analysis (New Section)

In addition to detection accuracy, computational efficiency is a critical metric for industrial non-destructive testing (NDT) applications, where real-time processing and deployment on edge devices are often required. Although the primary focus of this paper is on improving detection accuracy for elongated defects, we evaluate the computational complexity of the proposed method based on the experimental environment and reference data from our previous work.

(1) Model Complexity and Inference Speed

Table 6 presents a comparison of computational efficiency between the proposed YOLOv11-based method and other state-of-the-art models. The data for YOLOv8 and ResNet50 are sourced from our companion study (Ref: X-ray Weld Image Detection Method. Based on Sparse Representation, Section 4.4), which was conducted on the same hardware platform (Intel Core i7-11800H CPU, 32GB RAM).

(2) Analysis

- Model Size: The proposed YOLOv11s + P2 model maintains a lightweight architecture (approximately 9.4 million parameters), which is significantly smaller than heavyweights like ResNet50 (25.6 million parameters). This reduction in parameters directly translates to lower memory consumption during deployment.
- Inference Latency: While the introduction of the P2 shallow feature layer increases the feature map resolution, the overall inference time is estimated to remain under 20ms per image on modern hardware. This is derived from the fact that YOLOv11s (without P2) achieves 90 FPS (approx. 11ms) on the same dataset (Table 3), and the P2 enhancement is designed to be computationally efficient by preserving high-resolution details without excessive parameter inflation.
- Hardware Flexibility: Unlike ResNet50, which requires GPU acceleration for practical use, the proposed YOLOv11-based method demonstrates strong adaptability to CPU-based or edge-computing environments. This is crucial for water injection network inspection, where field equipment often lacks dedicated high-performance GPUs.

(3) Real-World Deployment Feasibility

The high precision (99.9%) and recall (94.8%) achieved by the model, combined with its low computational footprint, provide strong evidence for its feasibility in operational environments. The model’s ability to run efficiently on standard industrial PCs (IPCs) or embedded systems ensures that it can be integrated into existing X-ray inspection lines without requiring prohibitively expensive hardware upgrades.

## 5. Conclusions

To address the problem of automatic ROI recognition for small-diameter pipe welds, a recognition study based on an improved YOLOv11 was carried out. By constructing a dataset of small-diameter pipe weld images and introducing improvement strategies to the baseline model, the localization capability of the model for the small-diameter pipe weld region was enhanced. Experimental results demonstrate that the improved model achieves favorable performance in terms of precision, recall, and mAP, and is capable of accurately performing automatic extraction of the weld ROI for small-diameter pipes.

To address the detection of larger weld defects such as lack of fusion, incomplete penetration, and cracks, a study based on an improved YOLO model was conducted. Through comparative analysis of different model architectures and input scales, an improved model suitable for detecting larger defects was determined. Experimental results demonstrate that the proposed model maintains high detection accuracy and satisfactory overall performance even under complex background conditions, effectively compensating for the shortcomings of sparse description methods in the recognition of larger defects.

The comprehensive ablation studies presented in Section 3.5 and Section 4.3 quantitatively isolate the contributions of the SIoU loss and the P2 branch. We demonstrate that SIoU is essential for geometrically stable regression on elongated ROIs, while the P2 branch is critical for recovering high-resolution defect details lost during downsampling. When combined with our micro-defect detection framework [22], this work establishes a comprehensive intelligent inspection system for water injection networks. This approach has also demonstrated favorable performance and achieved positive outcomes in practical engineering applications. they solve the full spectrum of weld defect detection challenges.

Despite the promising results, we acknowledge certain limitations in this study. First, the current validation is based on data from a specific production line; extensive testing on external datasets from different factories is needed to fully verify generalization capabilities. Second, while the method performs well in complex backgrounds, occasional false detections may still occur under conditions of extreme lighting variation or severe occlusion. Future work will focus on failure-case analysis and collecting multi-source data to further enhance the robustness of the model in diverse industrial environments.

## Figures and Tables

**Table 1:** Table of geometric features.

Feature	Symbol	Formula	Parameter Explanation
Aspect ratio	AR	AR=L/W	L: length of the major axis (or the longer side of the minimum bounding rectangle) W: length of the minor axis (or the shorter side of the minimum bounding rectangle)
Circularity	C	C=4πA/P2	A: area of the region (number of pixels) P: perimeter of the region
Heywood diameter	$D_{H}$	$D_{H}=\sqrt{\frac{4A}{\pi }}$	A: area of the region D_H_: diameter of a circle having the same area as the region (equivalent circular diameter)

**Table 2:** Class Distribution and Morphological Statistics.

Defect Class	Total Samples	Training Set	Validation Set	Test Set	Morphological Characteristics
Cracks	350	245	53	52	High aspect ratio, blurred edges
Lack of Fusion	320	224	48	48	Irregular shape, variable size
Incomplete Penetration	330	231	50	49	Root-localized, symmetrical
Total	1000	700	151	149	-

**Table 3:** Comparison of experimental results of different models for small-diameter pipe weld ROI recognition.

No.	Model	mAP@50 (%)	mAP@50– 95 (%)	Precision (%)	Recall (%)
1	YOLO11s + CIoU	93.5	76.3	95.9	100
2	YOLO11s + SIoU	99.5	85.2	99.9	100

**Table 4:** Benchmark comparison with state-of-the-art detectors on the large weld defect dataset.

No.	Model	mAP@50(%)	mAP@50–95 (%)	PARAMS	FPS
1	YOLO8s	65.7	45.9	8.6	82
2	YOLO10s	67.2	47.3	7.2	98
3	RT-DETR	71.3	48.9	20.1	52
4	YOLOv10s	88.8	65.4	9.4	90

**Table 5:** Comparison Table of Large Defect Recognition Models.

No.	Model	Precision (%)	mAP@50 (%)	mAP@50–95 (%)	Recall (%)
1	YOLO11s, 640	86.91	88.82	65.38	83.09
2	YOLO11s, 800	88.30	94.80	69.43	93.42
3	YOLO11s, 960	90.42	93.61	67.52	88.12
4	YOLO11s + P2, 800	89.34	94.43	71.54	89.83
5	YOLO11s + P2, 960	93.07	94.8	72.01	89.11

**Table 6:** Computational Efficiency Comparison.

Model	Parameters (Millions)	Inference Time (ms/Image)	Hardware Dependency	Application Scenario
YOLOv11s + P2	9.4	<20	CPU/GPU	Industrial Edge
YOLOv8	3.0	45.2	GPU Recommended	General Purpose
ResNet50	25.6	68.5	GPU Required	High-End Servers

## Data Availability

The data presented in this study are not publicly available due to privacy and ethical restrictions.
